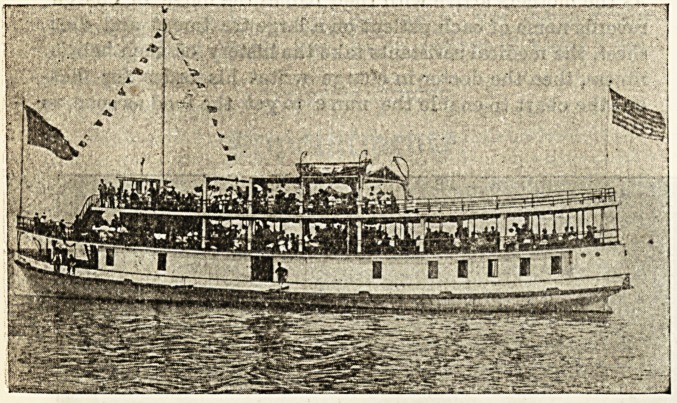# The Hospital. Nursing Section

**Published:** 1905-03-11

**Authors:** 


### The Hospital.

### Nursing Section.

Contributions for this Section of "The Hospital" should be addressed to the Editor, "The Hospital," Nursing Section, 28 & 29 Southampton Street, Strand, London, W.C.

No. 963.—Vol. XXXVII. *SATURDAY, MARCH* 11, 1905.

### Notes on News from the Nursing World.

### QUEEN ALEXANDRA'S NURSING SERVICE.

An important intimation has been made by the new Director-General of the Army Medical Service. Surgeon-General Keogh informs general officers commanding that it is considered desirable that matrons, or acting matrons, of Queen Alexandra's Imperial Military Nursing Service should keep a permanent record of all the sisters and staff nurses of the Nursing Service, and also of the Army Nursing Service Reserve members, who are serving under them in each hospital. The record is to contain the names of the members of the nursing staff and the addresses of nearest relatives; any special qualifications which they may possess, such as ability to lecture, to train orderlies in massage, electricity, and the light treatment, to nurse special diseases of women or children or midwifery cases; and also the dates of ordinary and sick leave, and of commencing and ending night duty. It is specially mentioned that the record is not to contain anything of a confidential nature, such as the character of the members or the manner in which they perform their duty.

### THE FATE OF THE NURSES' REGISTRATION BILL.

Even if the Parliamentary Session lasts the usual period, there is no chance that the Nurses' Registration Bill will be discussed before the prorogation. The measure is down for second reading on Friday, March 17th, but it is only the third Order for the day, and has before it two Bills of great interest, the Coal Mines Employment Bill and the Church Organisation and Discipline Bill, which are sure to be discussed at length. The Nurses' Registration Bill this year is in the hands of Mr. Munro Ferguson, husband of Lady Helen Munro Ferguson, and is backed by Dr. Farquharson, Mr. Haldane, Sir Thomas Wrightson, Sir James Joicey, Mr. H. C. Smith, Mr. Black, Mr. Eve, and Dr. Thompson. It consists of 25 clauses, and it is proposed by the projectors that, if passed, the Act shall take effect on October 1st, 1905.

### CARE OF INFANT LIFE IN AUSTRALIA.

The contrast between the social condition of Russia and our own Colonies at the Antipodes is illustrated in a striking manner by the short article in our columns to-day which deals with the question of Infant Life Protection in South Australia. In Russia, as another contributor showed recently, the lives of infants, legitimate or illegitimate, are practically of no account. But in South Australia, under the new Act, every infant separated from its mother is specially treated by the State, and consequently such scandals as have occurred in connection with baby farming here are rendered impossible. This is due to the fact that only one baby is allowed to each foster mother, and that the child is visited at regular and irregular intervals by an inspector who must evidently either be a trained nurse or a medical woman. In this respect we are behind Australia, but when, in due time, the State here undertakes similar responsibilities, there will be another opening for the employment of nurses who have no particular leaning to hospital or private work.

### TRAINING AT FEVER HOSPITALS.

In a lecture on "Some Practical Points in the Management of an Isolation Hospital," the medical superintendent of Monsall Hospital, Manchester, dwells on the vital importance of surgical cleanliness, and says that the extent to which it is possible must mainly depend upon the nursing staff. It is mentioned that at Monsall the nurse sterilises her hands by scrubbing them with soft soap and water, followed by the use of turpentine and methylated spirit. She then puts on a pair of rubber gloves, which have been recently boiled, and proceeds to the treatment of the patient. Afterwards the gloved hands are cleansed by holding them under the tap for a few minutes, followed by immersion in a solution of izal. Another precaution is that the nurse wears her sleeves rolled up above the elbow whenever she is doing anything for the patient. But, in order that the best results in nursing shall be obtainable, the medical superintendent at Monsall insists that it is essential to engage young women and train them, and this, he adds, is only possible when the hospital can give a certificate of training which is recognised by other authorities. Unfortunately, he says, it is the practice in some large fever hospitals not to engage junior nurses for any definite period, and then it is hopeless to attempt to secure conscientious work. At Monsall the junior nurses are practically probationers, and are trained for two years. The training consists in immediate supervision by sisters who have had a good general training of three years' duration, lectures on anatomy and physiology, and later, on the infectious diseases themselves, each course being followed by an examination. Small classes in practical ward preparation for operations and bandaging, are also held throughout the year, while at the end of two years a certificate is given. The fruits of the system at Monsall have been very satisfactory, and there is no doubt that a course of training on similar lines ought to be adopted in all fever hospitals which desire to be in the van of progress.

### TEN YEARS WAITING FOR AN ANNUITY.

The report of the Trained Nurses' Annuity Fund for 1904 shows a slight increase in the annual subscription; but it can be understood that more, very much more, than the £174 contributed in subscriptions and donations, is needed to meet the cases of the many applicants for aid. It is encouraging, however, to learn that the increase is due to the fact that early in the year the C uncil sent out a circular to every matron in the United Kingdom. Attention is drawn to the fact that the rules of the Association have been revised, the chief change being that the entrance fee now paid by nurses is reduced in proportion to their age. But what strikes us as by far the most significant item in the report is the announcement that a vacancy which has occurred by the death of an annuitant has just been filled by the election of a nurse, aged 71, who was approved as far back as 1894. If only when this poor woman was young the Royal National Pension Fund had existed she could have insured a sum sufficient to keep her in comfort when she was 50 years of age without that terrible decade of waiting!

### PLYMOUTH GUARDIANS AND THEIR SUPERINTENDENT NURSE.

Contrary to their previous resolution, the Plymouth Guardians have directed their clerk to draw up a report to be sent to the Local Government Board in reply to the demand for a statement of the charges against the superintendent nurse. This report was adopted unanimously. The principal charges against Miss Holliday are those of want of consideration towards the nurses under her, which it is alleged causes frequent resignations among the nursing staff. A charge nurse affirmed that when the night nurse went to report herself to the superintendent at 9.15 a.m. she was frequently kept waiting for 30 to 40 minutes, so that she lost her outdoor exercise; also that the superintendent refused any longer to allow the nurses' night meal to be served at 11.30 p.m. on Thursdays, instead of midnight. This arrangement was made so that the Roman Catholic nurses might have a good meat meal before Friday's fast began. The nurse also stated that though fish was served on other days, none was allowed on Fridays, and that Miss Holliday had especially requested a charge nurse to look after another nurse, whom she named, and "report what she could find against her." The view of the Guardians is that such incidents as these point to the desirability of a change.

### A PRACTICAL TEST.

The Committee of Newark Hospital have recently, at their own expense, installed hydrants and hose-pipes in the building. There is a resident surgeon, but he is the only male official in the hospital, and the action of the committee was criticised on the ground that the nurses would not be able to handle the hose. A few days ago a member of the Committee entered the hospital and raised the alarm of fire. In less than four minutes from that time the water was playing on the scene of the imaginary fire, which was at the extreme end of the building. The apparatus was installed, at the suggestion of the lady superintendent, a few months ago, and since then the nurses have had regular fire drill under her supervision. It must be admitted that they have emerged triumphantly from a practical test of their capacity to act as a fire brigade.

### PAUPER ATTENDANTS IN LANCASHIRE POOR-LAW INFIRMARIES.

The nursing return for 1905, which has just been issued by Mr. H. Jenner-Fust, general inspector for Lancashire and part of Cumberland and Westmorland, shows that the increase in the number of nurses has again just kept pace with the increased number of sick, but that there is still great need in several instances for more night nurses. The number of pauper assistants is 160, of whom 10 are employed at Blackburn Infirmary, 14 at Bury, 10 at Leigh, 22 at Prestwich, 10 at West Derby Mill Road Infirmary, and 12 each at West Derby, Belmont Road Branch and Cottage Home. At Whitehaven Infirmary there are also 10. It is true that ten years ago the number for the same district was 260, but as it went down gradually to 152 in 1902, the present position suggests the reverse of a progressive tendency.

### THE MORPHIA MYSTERY AT BATH.

In our later editions last week we were able to announce that the nurse who was accused of having attempted to poison the matron of the Bath Eye Infirmary was discharged at the police court. We have since learnt that the solicitor who appeared on behalf of the Director of Public Prosecutions made a statement of considerable length in which he said that he could not carry the case beyond the fact that morphia had been put in the glass of hot milk which the matron is in the habit of taking before she retires, though if the magistrates wished he would call evidence. We think that in these circumstances the magistrates wisely decided that no useful purpose would be served by proceeding with the case.

### EAST LONDON NURSING SOCIETY.

The annual meeting of the East London Nursing Society will be held next Tuesday at 3 p.m. at the Mansion House, the Lord Mayor in the chair. The annual report again shows a steady increase in the number of cases nursed and in the visits paid. The former amounted to 5,277 or 625 more than in 1903, and the latter to 123,218, or 8,009 more than in 1903. During the year three matrons and 27 nurses worked in the parishes, all the matrons and 10 of the nurses being Queen's nurses. For the first time for some years the society close the year's account in debt, and there is therefore all the more need that the response to the appeal which will be made next week should be generous. As the report was passing through the Press, Lord Malmesbury wrote asking to be relieved from the treasurership, and the Rev. H. V. Le Bas, preacher of the Charterhouse, who was chosen to succeed him, having offered to provide, in his own house, a room to be used as an office for the society, the offer was gratefully accepted. All communications should therefore be addressed to the Secretary, East London Nursing Society, Charterhouse, E.C.

### PRESTON INFIRMARY AND DISTRICT NURSING.

At a meeting of the subscribers to the Preston Royal Infirmary the other day, it was proposed that the board should engage nurses to visit the out-patients, or, in other words, establish a district nursing branch. It was said that an appeal to the public for funds for this purpose would meet with a liberal response. Probably it might, but as there are already trained district nurses at work in Preston, we do not think that it can be necessary or desirable for the infirmary to embark upon the experiment. If the sick poor are not sufficiently cared for under existing circumstances, the remedy would seem to be to augment the number of district nurses employed by the local association rather than to send out some from the infirmary.

### THE OBSTETRICAL SOCIETY OF LONDON.

The last examination of midwives by the Obstetrical Society of London has just been held. Out of 674 candidates 559 were awarded certificates. The society was established in 1872, and during the period of its existence no fewer than 12,805 candidates presented themselves for examination, 10,581 of whom passed. Henceforth all persons desiring to practise as midwives must pass the examination of the Central Midwives Board.

### A POPULAR MOVEMENT AT SWANSEA.

The movement started by the committee of the Swansea District Nursing Association of augmenting the receipts by a "penny a month" collecting scheme has been very successfully initiated. A great number of ladies have consented to serve as secretaries and collectors, and the reception accorded to the latter has been highly encouraging. Few have objected to give the penny asked for, and many have volunteered more. There is only one district in the town now without a secretary or collectors, and it is especially satisfactory to learn that those among whom the district nurses work are the most ready to respond to the appeal. It is hoped that the result of this effort will be to enable the Committee of the Association, whose receipts last year fell off to the extent of nearly £40, to at least refrain from drawing further from their small deposit account.

### A RECORD YEAR.

The report presented at the annual meeting of the Hammersmith and Fulham District Nursing Association shows that the number of cases attended during 1904, and the number of visits paid, far exceeded the figures for any previous year. The totals were 1,367 cases, with 29,861 attendances, as many as 87 visits being paid to one case, and 78 to another. Over 4,000 minor dressings, etc., were done in the schools, and 58 operations were attended. The returns for each month show that while March and November were the busiest months, the number of new cases was not less than 70 in any month, the average being 106. The work was accomplished on an income of £806 16s. 7d., £58 11s. of which was contributed by patients; £21 6s. granted by the Hospital Sunday Fund, £21 by the Boards of Guardians for the two parishes, and £10 10s. by the West London Police Court. While the area covered by the Association is over 16 square miles, with a population of nearly 233,000, the amount of local support is relatively very small, only £207 4s. being received in subscriptions, and £40 13s. in donations. There was a deficiency of £8 2s. 7d. on the year's working.

### AUCKLAND AND DISTRICT WORKING MEN'S NURSING ASSOCIATION.

The second annual report of the Auckland and District Working Men's Nursing Association which was adopted at the meeting last month, shows conclusively that the industrial classes continue to thoroughly appreciate the work of the organisation, and also that the labours of the nurses are increasingly heavy. Last year there were no fewer than 453 cases nursed, being an increase of 128 over the preceding year, while the total number of visits paid by the nurses exceeded 10,000. The sound financial position of the Association is attested by the fact that the balance in hand has risen from £24 to £64. The superintendent nurse, Miss V. E. Wood, who seems to enjoy not only the entire confidence of the Board of Management, but also, in a special degree the goodwill of the industrial classes, is unremitting in her attention to the sick. But as a good deal of her time is necessarily taken up in going to and from St. Helen's, a mining village close to Auckland, to look after the new branch, and as it is proposed to open out all the colliery villages in the vicinity her administrative duties will tend to increase. It is important that neither she, nor the nurses under her, should have to suffer from an undue strain upon their energy. The fact that nearly £100 was contributed to the association by means of subscriptions of a half-penny a week, while emphasising the importance of appealing to the many for small sums, justifies the conclusion that a further response may be obtained in the same quarters if more money is needed for another nurse. That some of the large employers of labour recognise the need of increasing the staff is proved by the fact that one firm has just promised £25 a year towards a nurse, on condition that their men contribute a halfpenny a week.

### SHORT ITEMS.

A social gathering was held last week at Marycourt, Bridgwater, when the ladies of the committee of the District Nursing Association entertained a number of nurses, and a lecture was given on "*x* rays" by Dr. Bird.—We understand that since the election announced in our issue of last week, of Miss West as charge-nurse at Bury Infirmary, a death in her family has prevented her from taking up the duties. The appointment is still vacant.—An enjoyable entertainment was given to the in-patients of the Cancer Hospital, Fulham Road, on Thursday evening last, under the management of Miss Lloyd Jones. The proceedings terminated with a hearty vote of thanks.—Miss D. V. Briscoe has resigned her appointment as sister in Queen Alexandra's Imperial Military Nursing Service; and Miss Ethel May Lang has provisionally been appointed staff nurse.—Miss Constance Clarke, who was married to Admiral Sir John Baird on Monday afternoon at St. Paul's, Knightsbridge, passed through a short course of training at St. Bartholomew's Hospital, and several nurses were present at the wedding.

### The Nursing Outlook.

"From magnanimity, all fear above;
From nobler recompense, above applause,
Which owes to man's short outlook all its charm."

### THE HIGHER EDUCATION AND TRAINING OF NURSES.

A NURSE writes:—"We are hearing the disadvantages of the Incorporated Society for Promoting Higher Education much discussed, and we feel there may be many benefits if we were only cognisant of them, and we really want more light on the subject. Is it too much to ask you to let us know how it will actually affect trained nurses, and will it eventually bring about State registration, or is this society to take the place of it?" These inquiries are very natural at the moment. The present position of affairs arises from the failure of the nurse-training schools to co-operate together with the object of securing the higher education and training of nurses, and from the circumstance, that, owing to this default on their part, a relatively few but active spirits, known as the stage army, are endeavouring to obtain the control of nursing affairs in this country, in the shadow of an agitation for the establishment of a system of State registration. Such a position of affairs would be lamentable in any event, and it is made immeasurably more difficult from the circumstance that the majority of women, who are working as nurses to the sick in various capacities, have obtained such training as they have, from no fault of their own, piecemeal, or under conditions which have never enabled them to procure a certificate of training which would qualify them to take their place as fully-trained nurses in the highest and best sense. We have shown over and over again, that much of the present confusion is created by this state of affairs, which causes a large number of women to pose as nurses and impose on the public as such, who have never been adequately trained, and who may in fact never have had any real training in nursing at all. Unless a plan can be devised which will enable every nurse possessing the necessary experience and knowledge to obtain a certificate of average efficiency, little effective reform is practicable.

The Incorporated Society for Promoting the Higher Education and Training of Nurses embodies a practical plan whereby the necessary reforms can be brought about by the simplest means. This society has applied to the Board of Trade to be incorporated under a clause in an Act of Parliament which forbids any of the members or signatories to the constitution and articles to make any profit of any kind out of the organisation. This provision gives an absolute guarantee to the nurses that all money which may accrue to the Incorporated Society will be devoted in its entirety to the sole purpose of promoting and improving the higher education and training of nurses. The Incorporated Society, if the Board of Trade grants it a license, will therefore be founded upon so pure and public-spirited a basis, that every nurse may have the most perfect confidence in using its machinery and organisation to help her in her work, and to continuously acquire such a knowledge of her profession, that she may be always able to hold her own in the nursing world, and command the best remuneration for her services.

The constitution of the Incorporated Society provides amongst other things that (1) its affairs will be under the control and management of a council consisting of heads of training schools, nurses, and medical men engaged in the teaching and training of probationers; and (2) that one chief object will be to offer facilities to all nurses who care to present themselves for examination, wherever they may have been trained, to show that they possess the knowledge and training which enables them to hold the Society's certificate as a trained nurse of character and competency; (3) to promote the higher education and training of nurses to the fullest extent possible. There is of course a number of other objects which the Society hopes to accomplish in the direction of post-graduate instruction and the strengthening and helping of nurses in various directions; but these need not detain us to-day. The one material fact is that a number of merchant princes have made themselves responsible for a certain sum to enable the Incorporated Society to be established with the object of helping all nurses of capacity and knowledge to take their proper place in the nursing world in England to-day, and so to gradually bring about the uniting of all interests in the cause of efficient nursing. In such circumstances, it must be difficult for those who are not acquainted with the methods and objects of the stage army and those who control it to understand, why any objection should be made to a society founded on so just and honourable a basis with such excellent objects. We have felt all along that the opposition is so unworthy and ridiculous as to call for little or no comment in these columns.

As to the effects such a society may have upon State registration we may point out that if it succeeds in uniting the majority of capable workers with character in the nursing world in England to-day upon a uniform basis of efficiency, it will place Parliament in a position to decide for or against State registration on its merits. Certainly some such intermediate plan as that formulated by the Incorporated Society must first clear the ground by securing justice for all nurses who are worthy, before Parliament is likely to grant State registration at all. This is a point which the advocates of State registration cannot or will not recognise, though in fact it constitutes the underlying cause which makes it practically certain that the State registration of nurses is not likely to be set up by Act of Parliament in this country for many years to come.

### Lectures upon the Nursing of Infectious Diseases.

By F. J. Woollacott, M.A., M.D., B.S.Oxon, D.P.H., Senior Assistant Medical Officer, Park Hospital, Metropolitan Asylums Board, Hither Green.

LECTURE XVI.—SMALL-POX (*Continued*).—VACCINATION—CHICKEN-POX.

We have already dealt with some of the complications of small-pox that arise in connection with the rash, and have seen that for the most part they result from the spread of inflammation from the pocks to the surrounding structures. A few others remain to be considered.

More or less bronchitis is nearly always present, if the case be at all severe, and more extensive inflammation of the lungs may follow. In confluent attacks, during the stage of secondary fever, pneumonia is by no means rare, and is sometimes associated with pleurisy which may become purulent.

Occasionally suppuration of one or more of the joints occurs, usually during the scabbing-stage of a severe eruption. The joints most often affected are those immediately under the surface such as the outer end of the collar-bone and the elbow, and probably in these cases the inflammation spreads directly from the deeper layers of the skin.

As in many other of the infectious diseases the action of the kidneys may be impaired, and albuminuria is of frequent occurrence. Acute nephritis, too, is not uncommon, and in this respect small-pox and scarlet fever resemble one another. In both diseases the nephritis begins somewhat late, in one case in the scabbing and in the other in the peeling stage.

The other complications of small-pox are comparatively rare, and need not be referred to here. They all require precisely the same treatment as when due to other causes.

If the patient be pregnant the risk of abortion or premature labour is considerable, and in confluent or hæmorrhagic cases it is almost certain to take place. The bleeding, both before and after delivery, is often excessive. The child, even if born alive, usually dies in a few days, and, as it has already been infected by the mother, may develop the disease in spite of vaccination. As a rule, after small-pox, recovery is complete in every way, though after a severe and complicated attack, convalescence may be slow and tedious. Permanent scarring of the skin, however, is nearly always left, the only exceptions being the very slight cases with scanty and modified eruption. After confluent attacks the face is often greatly disfigured, especially if it has been much torn or scratched. The scars are usually depressed and pit-like, and, though they ultimately become white, may remain of a reddish-brown colour for weeks. In some cases the scars are raised above the level of the surrounding skin and an irregular, warty appearance results. The hair in confluent attacks often falls off, leaving the scalp almost bald, but sooner or later it grows again. The most serious after consequence of the disease is loss of sight in one or both eyes, resulting from ulceration of the cornea.

A case of small-pox continues to be a possible source of infection until all the scabs have separated. The hands and feet are the last parts of the body to clear, for, owing to the thick skin of the palms and soles, the pustules do not rupture, but dry up into small, round, brownish "seeds." These are picked out and removed by cutting through the skin with scissors; and when the nurse is doing this she should not forget to examine between the toes, for a seed in this position might easily escape observation unless sought for.

While in attendance on small-pox patients the nurse should never lose sight of the intensely infectious character of the disease. She need have no anxiety about herself, for, if she have been recently and efficiently vaccinated, she is quite safe; but she should take the most careful precautions against carrying the infection outside the hospital, and she should remember that every article brought into the ward soon becomes saturated with infection and therefore capable of spreading the disease. If she be going out she should never wear any single article of clothing that she wears when on duty, and she should take a hot bath, not omitting to thoroughly wash the hair. In some small-pox hospitals a set of rooms is provided to enable these precautions to be properly carried out. In one room the nurse leaves her infected clothing, in a second takes a bath, and in a third, which is kept free from infection, puts on her out-door clothing.

### Vaccination.

By vaccination is meant the inoculation of the skin with lymph derived either from a previous case, or, as is more usual at the present time, from a calf suffering from cow-pox. As a rule the lymph is kept stored in little glass tubes. When wanted for use the ends of a tube are broken off, and the contents are blown out on to a part of the skin, usually of the upper arm, which has previously been lightly scratched, and then well rubbed in and allowed to dry. In the course of a few days the places begin to itch and become slightly inflamed. Little papules then appear, which by the end of a week have developed into vesicles containing a clear fluid. A little later the fluid becomes thick and cloudy, oozes out and finally dries up into a scab, which on separating leaves a scar usually more or less studded with a number of tiny depressions.

At the time of vaccination, and afterwards, the same precautions should be observed as in any other operation. The arm should be thoroughly cleaned, and any instrument that may be used sterilised by heat. A simple dressing, such as sterilised wool or gauze, should afterwards be applied and lightly bandaged on. It need not be disturbed for some days. A certain amount of inflammation and itching is inevitable, but with a reasonable amount of care there is no danger, and no harm will result, and it is well worth while enduring a little discomfort in order to obtain protection against such a horrible disease as small-pox. If the wound is allowed to get dirty, or irritated by a tightly-fitting sleeve or otherwise, then all sorts of trouble may follow.

A successful vaccination is for a time an absolute safeguard against small-pox, and, even after the lapse of that time, though its influence may not be strong enough to ward off an attack, it is frequently able to modify it and prevent it from becoming severe. Full protection is easily regained by repeating the vaccination. In this country vaccination in infancy is compulsory by law, and, as its influence is not permanent, it should be a routine practice to repeat it at about ten or twelve years of age. After exposure to the infection of small-pox it should be always repeated if necessary, for it will be able to prevent an attack of the disease when successfully performed within three days. If delayed beyond that time it may be of little or no use, and in such cases it is not unusual to see the vaccination and the small-pox eruption developing side by side.

### Chicken-Pox.

Chicken-pox, or varicella, is an infectious disease attended by a characteristic vesicular eruption. It is almost invariably of a mild character, and without danger to life, and its chief importance arises from its resemblance to small-pox, for which it is not infrequently mistaken. For the most part it is met with in young children, and probably most people suffer from it at some period of their lives. The infection usually spreads directly from person to person, and occasionally by the agency of clothing and similar articles. The incubation period is a long one—from 12 to 19 days.

The early symptoms are vague and indefinite. There may be a slight rise of temperature, and perhaps the patient may be rather fretful or restless, or a faint erythematous rash, not unlike that of scarlet fever, may come out on the trunk. Very frequently, however, the first sign of the disease is the appearance of the characteristic eruption, which develops very rapidly, so that its early stages often escape notice. Each pock begins as a little red or pink spot, which soon becomes raised up into a papule. In a few hours it becomes distended with clear fluid, and is converted into a vesicle. These vesicles are somewhat oval in shape, with a rounded top, and about the size of a split pea. As a rule there is very little surrounding inflammation, so that they look not unlike drops of water on the skin. They soon rupture, their contents escape and dry up into small scabs, which on falling off may leave more or less well marked scars. The eruption comes out in crops, taking four or five days or more; consequently it is common to see, on the same part of the body, pocks in different stages of development—scabs, vesicles, and papules all being mingled together.

The rash begins on the trunk, where it is nearly always most marked; but the head, face, and limbs are soon involved. It is rarely very abundant, and never becomes confluent, as so frequently happens in small-pox. On the arms and legs the full development of the pocks is often arrested, so that they do not become vesicular, but appear as small, round papules, firm and resistant to the touch. Their appearance then very closely resembles that of the pocks in modified small-pox. As a rule a few vesicles form on the mucous membrane of the mouth, and may be seen on the soft palate as little, round, slightly inflamed ulcers.

During an attack of chicken-pox the patient rarely experiences much discomfort; and, indeed, apart from some irritation and itching in connection with the pocks, often feels quite well in every way. He should be kept in bed for a few days till the whole of the rash has appeared, and till the temperature has fallen; and during this time it is usual to restrict the diet somewhat, though in ordinary cases this is hardly necessary. No special treatment is, as a rule, required for the eruption, and the scabs fall off spontaneously. As far as possible scratching should be prevented, if necessary by muffling the hands or fixing the arms in splints, so that the elbows cannot be bent. If this precaution be neglected deep scarring may result, or the pus may be carried under the finger nails to different parts of the body, and give rise to troublesome sores. Occasionally a pock becomes considerably inflamed, or perhaps ulceration may occur under the scab. In that case healing will be facilitated by softening the scab with carbolic oil and applying poultices or boracic fomentations. If there be much rash on the scalp it is advisable to cut the hair short. During convalescence baths should be given each day. The mucous membrane of the mouth is usually very slightly affected and soon heals. If there be any discomfort a mouth wash, or painting with glycerine of borax, will afford relief.

The complications of the disease are not important, and apart from a few scars, which are rarely very deep, there are no after consequences.

In some very exceptional cases the pocks may become the seat of a gangrenous process. They enlarge, and become much inflamed, and the tissues under the scab begin to slough. The patient is usually ill-nourished and unhealthy, and very frequently dies. The treatment consists in maintaining, as far as possible, the patient's strength by a nourishing and plentiful diet, and removing the scabs and sloughs by poultices and antiseptic dressings.

The infection of chicken-pox persists until all the scabs have fallen off. As is the case in small-pox, the pocks on the feet sometimes do not rupture, but dry up into brownish "seeds." These should be removed by cutting through the overlying skin.

### Infant Life Protection in South Australia.

BY OUR OWN CORRESPONDENT.

ALTHOUGH South Australia is one of the smallest of the States of the Commonwealth, in the matter of infant life protection it has been a pioneer in a large number of experiments. This has notably been the case with the juvenile court movement. Improving upon the Massachusetts plan of separate hearings in children's cases, South Australia has had its legally constituted Children's Court—the first in the world—at work in Adelaide as far back as 1890.

The South Australian baby is not too highly favoured in his surroundings. The long hot summer is trying to the strongest infants, and is apt to tell cruelly on the hand-fed child on account of the difficulties in the way of keeping milk sweet. And that child in the community upon whom unfavourable conditions tell most quickly is undoubtedly the illegitimate child, and especially the illegitimate child whom its mother has had to board out. The licensing of the nurse who undertakes the care of young children for payment is a common provision, but, as far as I know, South Australia has been the first to carry the principle of official oversight of such nurses out in logical completeness.

I spent a day and a half not long ago accompanying the woman inspector on her rounds in and about the city of Adelaide. No study of Acts of Parliament and books of regulations could have convinced me as did the evidence of my eyes that it is possible to insure for these little ones such a high standard of care. To begin with, the professional nurse who has to make her living out of the children is practically non-existent. The rule which allows only one baby to each nurse, unless in exceptional cases, brings it about that it is the decent working man's wife or daughter who becomes the infant's foster-mother. She can make with the baby's own mother any business arrangement she pleases, and the State Children's Department will not interfere. It merely stands there as an interested on-looker to make sure that the baby does not suffer. Through its inspector, Miss Moule, the department must first of all be sure that the home is a good one, clean and well-kept, and the occupants respectable. Otherwise the license is refused. The license can also be cancelled at any time for all sorts of reasons. The wise provision that only one foster-infant is allowed in one home unless there be some one besides the foster-mother—say a grown-up daughter or younger sister to help—means that the baby is treated like the baby in an ordinary family, the centre of a little wholesome attention, coming in for the odds and ends of time of the bigger children as Nature surely meant every baby should do. A clean home, a kindly mother, and a reasonable prospect of attention offer the child a fair chance. Next it is the inspector's care to see that he thrives. If not she has to set her wits to work to find out why not. The foster-mother is furnished with printed instructions as to the times of feeding and the quantity to be given. If milk is not assimilated the foster-mother may have to try other foods, and possibly obtain medical advice. The feeding-bottles—which must have no long rubber tubes—must be kept scrupulously clean, and when not in use must lie in clean water with a pinch of baking-soda, for the inspector may drop in at any moment. She visits every child at least once a fortnight, and sometimes if a baby be ailing twice in one day. A woman of tact and experience will not be looked upon as a mere fault-finder, but rather as a helper and an adviser in difficulties.

When it is remembered in what a condition these children often are before being received by the nurses, suffering from the effects of drugs taken by the girl-mother before the birth, afflicted with congenital disease or half-starved for the first three or four months before the mother could make up her mind to part with her infant, it will be understood that that plan must be a good one which has reduced the death-rate from 18 per cent. to an average of about 8 per cent. per annum.

### Appointments.

[No charge is made for announcements under this head, and we are always glad to receive, and publish, appointments. The information, to insure accuracy, should be sent from the nurses themselves, and we cannot undertake to correct official announcements which may happen to be inaccurate. It is essential that in all cases the school of training should be given.]

Borough Isolation Hospital, Leicester.—Miss C. M. Duffy has been appointed matron. She was trained at the Royal Albert Edward Infirmary, Wigan, and has since been head nurse at the Park Hill, Hospital, Liverpool, and night superintendent and assistant matron at the Lodge Moor Hospital, Sheffield.

Chelsea Hospital for Women, London.—Miss Amy Sharp has been appointed charge nurse. She was trained at the Middlesbrough Sanatorium, where she afterwards became charge nurse, and at the Royal Albert Edward Infirmary, Wigan, where she has since been sister.

Cheshunt Isolation Hospital.—Miss Nellie Chapman has been appointed sister. She was trained at the Western Fever Hospital, Fulham, London, and has since been on the staff of the Nurses' Home, Doncaster.

Cotswold Convalescent Home, Cleeve Hill, near Cheltenham.—Miss Alice Green has been appointed lady superintendent. She was trained at the Nightingale Home, St. Thomas's Hospital, London. She has since been sister at the York County and Wolverhampton General Hospitals, and matron at Bradfield College Sanatorium and the Homes of St. Barnabas, East Grinstead. She has also done private nursing in Oxford, Bath, and London, and district work in Cheltenham.

Cottage Hospital, Westbury, Wilts.— Miss Dora Lush has been appointed nurse matron. She was trained at the Radcliffe Infirmary, Oxford, and then joined in succession the Trained Nurses' Home at Bath, the Cottage Hospital, and the District Nursing Institution at Brixham, South Devon.

Edinburgh Royal Infirmary.—Miss Alice Watson has been appointed masseuse. She was trained in London, and has since been practising in Edinburgh.

Hospital for Infectious Cases, Birkdale.—Miss Elizabeth Hughes has been appointed staff nurse. She was trained at Salford Union Infirmary.

Incorporation Infirmary, Shirley Warren, Southampton.—Miss Gertrude Eeles has been appointed sister. She was trained at the Poplar and Stepney Sick Asylum and has since been staff nurse at the Royal Infirmary, Sheffield, and has done private nursing in connection with the Royal United Hospital, Bath. She holds the L.O.S. certificate.

Isleworth Infirmary.—Miss Edith A. Hood has been appointed night superintendent. She was trained at Birkenhead Infirmary, where she was afterwards sister. She has also been ward sister and theatre sister at Bethnal Green Infirmary, London. She holds the certificate of the London Obstetrical Society and the Central Midwives Board.

Newbury Free Church Council.—Miss Eva Coome has been appointed district nurse. She was trained at Bristol Royal Infirmary, and has done district nursing in Barry and Cardiff.

St. Pancras Infirmary (South).—Miss Lilly Bentley has been appointed nurse-matron. She was trained at Fusehill Hospital, Carlisle, and St. Olave's Infirmary, Rotherhithe, where she was afterwards charge nurse, second assistant matron, and first assistant matron.

Victoria Hospital, Blackpool.—Miss Edith Brown has been appointed sister. She was trained at Burton-on-Trent Hospital, and has since been staff nurse at the Chelsea Hospital for Women, and at the Royal Infirmary, Sheffield.

Withington Infirmary, Manchester.—Miss Ethel P. Short, Miss May Costain, and Miss Louisa Wolstenholme have been appointed staff nurses. Miss Short was trained at Poplar and Stepney Sick Asylum, Miss Costain at Tunbridge Poor-law Infirmary, and Miss Wolstenholme at Barton-upon-Irwell Poor-law Infirmary.

Workhouse Infirmary, Gravelly Hill, Birmingham.—Miss Frances H. Brettell has been appointed home sister. She was trained at Mill Road Infirmary, Liverpool, and was afterwards night superintendent at West Ham Infirmary, London.

### Death in Our Ranks.

We regret to hear of the death of Miss P. J. Brady on March 5th. For nearly four years she had been matron of the Pembroke Cottage Hospital, and was formerly nurse at Stratford-on-Avon Hospital.

### TRAVEL NOTES AND QUERIES.

By our Travel Correspondent.

Accommodation in Amsterdam (Berrie).—Pension Bellevue in the Sarphatistraat, is fairly reasonable and in a good situation. Exact terms I do not know. I think you would do well to go to a moderate hotel, where bed and breakfast would be, roughly speaking, 3*s*. Many hotels charge one and three-quarter florins for that accommodation, which is as near to 3*s*. as we can reckon. As you will probably be out all day and frequently far away from your temporary home at meal times, it will prove more economical to have dinner wherever you may be at the time. Hotel Haas, at the corner of the Papenbrug steeg; Hotel Oldewelt, 100 Nieuwendyk, and Hotel-Restaurant Frank, 95 Damrak, are all reasonable as Amsterdam prices go, but you must remember Holland is not a cheap country for tourists.

Rules in Regard to Correspondence for this Section.—All questioners must use a pseudonym for publication, but the communication must also bear the writer's own name and address as well, which will be regarded as confidential. All such communications to be addressed "Travel Correspondent, 'Nursing Section of *The Hospital*,' 28 Southampton Street, Strand." No charge will be made for inserting and answering questions in the inquiry column, and all will be answered in rotation as space permits. If an answer by letter is required, a stamped and addressed envelope must be enclosed, together with 2s. 6d., which fee will be devoted to the objects of "The Hospital" Convalescent Fund. Ten days must be allowed before an answer can be published.

### The Boston Floating Hospital.

By L. A. WILBER, Superintendent of Nurses.

The Boston Floating Hospital had its beginning ten years ago, and is designed solely to give infants ill with gastro-intestinal and other non-contagious diseases the best of modern medical and nursing care. Most charitable organisations begin in a small way, and the Floating Hospital is no exception, for during the first season, in 1904, only two trips were made. The boat, which was just a picnic barge, towed by a tug, was hired, and such things as were needed for the day—cots and hammocks for beds, food, medicine, clothing, bathing appliances, etc.—were carried aboard; then at night the things that could be used again were packed and stored till the next trip. With such means but little systematic or scientific work could be done, but it was a beginning. Large numbers of mothers came and brought their babies, who were under observation from 9 A.M. to 4 P.M. In 1897 the managers were able to purchase the boat, and to have it fitted for hospital work, with bathrooms for hot and cold salt water baths, and a small operating room. Wards A, B, C, and D were established, thus making it possible to care for babies day and night. Since then the permanency of the work being assured, all departments of the work have constantly improved and satisfactory results have followed.

### A Unique Hospital.

In caring for sick babies night and day I believe the Boston Floating Hospital to be unique, other floating hospitals for infants caring for their patients only during the day. The boat is in commission from July 5th to September 15th, making about ten weeks of actual service. For accommodation of permanent patients 57 beds are available, and the top deck protected by awnings overhead is used for day patients only, and can accommodate 100 or more babies with their mothers, these being on the boat from 9 A.M. to 4 P.M., as at first. It is frequently found desirable that babies who are seriously ill should become in-patients, then they do not return at night to their homes.

### Scene on the Wharf.

A goodly amount of work is done on the wharf before the arrival of the boat from its night quarters in East Boston. As the mothers arrive with their babies the nurse in charge records name of each patient on a large treatment and diet sheet, the medical assistants take the history of each baby's illness, then the doctor in charge writes his order for diet on the chart to enable the nurse to get the food as soon as the boat arrives. About ten minutes after the patients are aboard each baby is provided with the special food its condition requires. To do this for so large a number of babies and so soon after their arrival, it is, perhaps, needless to remark that we require a perfect system and a first class food department, both of which we are fortunate in possessing. It is not uncommon on a very hot morning for the out-patient nurse to find waiting on the wharf a very ill baby, or a baby in collapse; so she provides herself with medicine for emergencies that the seriously ill baby need not wait to be really on the boat before treatment is begun; this means, of course, a saving of time in the baby's favour.

### The Appearance of the Boat.

In appearance the boat is rather quaint, being quite unlike the other craft one sees in the harbour, and we are still towed

**THE BOSTON FLOATING HOSPITAL**—*Continued.*

by a tug, as at first, to avoid the jar and discomfort of propelling machinery. The *Leader*, as the tug is called, tows its precious freight about eight miles down the harbour and anchors for a few hours, then slowly steams back to City Wharf, where day patients are discharged, many receiving return cards to enable them to be benefited by as many days' treatment as is thought necessary by the physicians. From the first the attitude of the hospital toward the mothers has been one of utmost consideration; they can come to inquire as often as they wish, and can stay aboard and make the trip if they desire, mothers of out-patients assisting in the care of their babies. They are allowed to bring one well child if they cannot leave it at home, and both are given a substantial lunch at midday. For these well children a kindergarten is arranged. The mothers receive instructions regarding the care of babies; what to do at the beginning of illness, and the preparation of food, and each mother receives special instructions as to feeding when she takes the baby home. Because of these privileges and because they see so much of the work, the mothers are quite willing to leave their babies whenever they require the continuous care given in the permanent wards.

Manipulating the Atmosphere to Suit the Babies.

A unique feature of two of the permanent wards is the atmospheric plant for changing the quality of the air for the hospital patients. The necessity for this plant was shown during the season of 1898, which was very severe, when one of the distressing features of the work was the disastrous result of the hot sultry days of August. On such days a whole ward full of patients would grow markedly worse and in many cases a fatal issue could be directly traced to unfavourable weather conditions. The object of the plant, to quote from a physician, is "to bring October weather into dog days," or in other words taking the air in its varying conditions of temperature and percentages of moisture to reduce its relative humidity to about 50°, and to raise or lower its temperature to about 74° F., which has seemed to be about right for hospital wards for babies. To illustrate: on August 8th when the natural air conditions were average temperature 69° F., average relative humidity 88°, the wards were kept at relative humidity of 56° and an average temperature 74° F. Of course, the results showing the success of the plant can not be calculated in figures, but there is no doubt that many of the babies owe their lives to the cool dry air furnished them when the natural air conditions were unbearable. From the time the boat was owned constant effort has been made to make the work of educational value, not only to the mothers but to young physicians and to nurses as well.

(*To be concluded.*)

### An Examination Nightmare.

My wits are gone to gather wool,
I cannot think or write,
And every simple, well-known fact
I knew has vanished quite.
My information's all astray,
My "bones" are all misplaced;
The "circulation's course" I'm sure
Is utterly effaced.

What shall I do? The minutes fly,
My brain seems all on fire.
I cannot find a thing to say
In spite of hot desire.
Why did I not take better notes?
Why did I scamp the work?
And why those nerves with awful names
Did I of malice shirk?

Elusive, now, they playfully
In numbered sequence stand,
And though they dance before my eye,
Elude my trembling hand.
I'll let them dance! I'll try the "spine."
I must remember some
Of all that list—a blackboard full,
And yet, not one will come!

Oh, would I were Ezekiel
To make these dry bones live!
What answers to those questions then
I should have power to give.
But Remington's prosaic type
Stares blankly from the page.
Unlit by fire from ancient days,
Or light from modern sage.

The pens of ready writers seem
To scratch on every side.
Their pages fill with flowing words,
A never-ebbing tide.
While on my foolscap's pallid face
No dawn of thought appears,
'Tis only sullied in one place
By ineffectual tears.

O shades of all the lectures that
I've ever listened to!
Attend, ye nine, or maybe twelve,
And hearken to my woe.
I can't recall the joints and bones
Of my anatomy;
Nor plasma, tissues, organs, cells,
Of physiology.

So I shall fail, I know I shall,
And when the list comes out,
Down at the foot—"To attend again"—
I'll be, without a doubt.
But what is this? I hear a bell,
And half asleep I seem—
Why, true it is that after all,
It's nothing but a dream!

E. M. F.

### Everybody's Opinion.

[Correspondence on all subjects is invited, but we cannot in any way be responsible for the opinions expressed by our correspondents. No communication can be entertained if the name and address of the correspondent are not given as a guarantee of good faith, but not necessarily for publication. All correspondents should write on one side of the paper only.]

### TRAINING AT A NURSING INSTITUTE.

MISS HEATLEY, matron of St. Clement's Maternity Home and Nursing Institution, Fulham, writes: Will you kindly allow me to state that the "Fulham Nursing Institute and Midwifery Training School" has no connection with this institute.

MISS WORRALL, matron of the Fulham Midwifery School, St. Mary's Nursing Home, 41 Parson's Green, Fulham, S.W., writes:—In your report on "Training at a Nursing Institute," you refer to the place as the "Fulham Nursing Institute and Midwifery Training School." If you will look at your advertisements you will see that that is the name and title of this institution, and I may remind you that it has been known to you by that name for many years. Such a report will naturally be attached to the home bearing that name, and is calculated to do it very much injury. Will you kindly therefore draw attention in your next issue to the fact that the institution in Munster Road to which the paragraph refers is not, and has no connection with, the Fulham Nursing Institute and Midwifery Training School.

### WOMEN INSPECTORS OF MIDWIVES.

"A. M. R." writes: I have been much interested in the letters from "W.," "Constant Reader," and "L. M. B." on the subject of Inspectorships of Midwives, and I am in entire sympathy with the two former correspondents. I was trained in one of our largest general hospitals, also in a fever hospital, in district nursing, and I have taken the L.O.S. certificate. Thus I have had experience in both branches of the profession, and I fail to see why general training should be considered a *sine quâ non* for an inspectorship of midwives. Also anyone who has been a practising midwife in a thickly-populated district as I have, with 2,355 cases in eight years, must of necessity have had more practical experience than a newly-qualified medical woman who cannot possibly realise the difficulties and conditions generally with which a district midwife has to contend. I would like to remind "L. M. B" that there are many chances for promotion in the general nursing world, but an inspectorship was the sole "rise" that a midwife could reasonably expect to get before the most recent disappointments were made known.

"QUALIFIED MIDWIFE" writes: In answer to "L. M. B.'s" letter I fail to see where the injustice comes in of employing other than three years' trained nurses for responsible posts as district nurses. I am sure that no committee, or person responsible, would engage a district nurse unless she was capable of fulfilling the duties required of her. When there is an appointment vacant certainly the fully-qualified nurse stands much the best chance of gaining that appointment; but if the insignificant maternity nurse is fortunate enough to secure it, well, who can blame her for doing so, and why, then, should she not receive the same salary? In my humble opinion it is not the length of time a woman is in a hospital that makes a nurse of her.

### DISTRICT NURSING IN MIDWIFERY.

"DISTRICT NURSE" writes: Will you allow me to make a few remarks on combining district nursing with midwifery: If all district nurses combining midwifery with general nursing worked under rules similar to those provided by our nursing committee I do not think that there would be any element of danger. We entirely separate bags, instruments, etc, visit maternity cases always first, and do not attend any infectious cases or enter an infected house knowingly. If one of us unfortunately does so she can call in the medical officer of health and the nurse provided for such cases, thoroughly disinfect herself and subject herself to the quarantine prescribed by the medical officer. In all cases our nurses must carry out the London Obstetrical Society's rules especially Section B 1 and 2, which are similar to those laid down by the Central Midwives Board in their instructions to midwives. I contend that the maternity patient under these circumstances is better protected than a patient attended by a doctor where a Gamp is employed, who may have come from a house reeking with infectious material, and where none of the necessary precautions or rules are even known, much less carried out. With regard to laying out the dead, there are generally women in the village capable of undertaking that office, with the undertaker's assistance. If a midwife is not to do general nursing, how is she to fill up her time in these rural districts? Supposing she has a dozen to 20 cases in the year, the only available sources of income in a village would be shop-keeping, dress-making, or plain needlework, and would not these entail as great a risk of infection as the general nursing? Would her hands be in a fit condition, and would not this be returning to the old Gamp system?

### TESTIMONIALS BY GUARDIANS' COMMITTEES

INTERESTED writes: It often occurs that testimonials (so-called) granted to fully-qualified ward sisters or charge-nurses resigning their posts are the cause of much friction between the recipients and the Boards of Guardians by whom such certificates are issued. In the case of union infirmaries there are generally two or three Guardians appointed as a visiting committee—busy people, who usually have no knowledge of infirmary administration and discipline, the management of sick people, or the training of probationers, though they give ready ear to unfounded complaints without inquiring into evidence. Such visitors, having a very imperfect acquaintance with the work of ward sisters or charge nurses, yet presume to pass judgment on their duties by granting a testimonial for good service in terms which the recipient is generally ashamed of. The opinion of the infirmary matron, who really knows what expression is deserved, is entirely dispensed with in the official testimonial, and I venture to say that the certificate of ability resulting from the Guardians' deliberations is most often such as any respectable guardian would personally be sorry to give to a doubtful employée leaving his service. Surely when testimonials carry weight in many directions there should be some remedy for this evident injustice! And the remedy seems to be that Guardians should keep to their own sphere of election and leave the drafting of testimonials to the matrons they appoint who can at least represent the truth in the reports of their assistants.

### Presentations.

MANCHESTER SOUTHERN HOSPITAL.—Mrs. Elène Wilkie, on leaving the Southern Hospital, Manchester, has been presented by her committee with a purse containing £20, a travelling bag from the nursing staff, and knives, forks and spoons from the servants. She has been for 12 years lady superintendent, and is leaving to take up work in London.

SWANAGE COTTAGE HOSPITAL.—Miss Annie Goldsmith, district nurse, has been presented, upon leaving the town of Swanage for South America, with a purse containing 13 sovereigns from the people amongst whom she has worked for the past eight years.

### Where to Go.

THE DOWDESWELL GALLERY.—A most interesting exhibition of water-colours, illustrating old-world gardens in England, Scotland, and Italy, is on view at the above galleries, 160 New Bond Street. The pictures are by Mr. E. Arthur Rowe.

### Echoes from the Outside World.

### Movements of Royalty.

On Monday Prince Ferdinand of Bulgaria arrived in London on a visit to the King and Queen at Buckingham Palace. The Prince is a member of the Saxe-Coburg Gotha family, being the eldest son of Prince Auguste and Princess Clementine of Bourbon-Orleans, and therefore a grandson of Louis Philippe. He was born in 1861, and in 1893 married one of the Princesses of the House of Bourbon-Parma, who pied in 1899. The visit is of a private nature. It is announced that the Queen, Princess Victoria, Prince and Princess Charles of Denmark will visit Lisbon, starting probably on Monday next.

### Manifesto by the Tsar.

In a Manifesto issued on Friday morning last the Tsar calls on his people to rally round the throne and says that Providence has visited Russia with heavy trials. A sanguinary war in the Far East, the honour of Russia, and the command of the Pacific Ocean, so urgently necessary for the consolidation of the prosperity not only of their own but of other Christian nations, have imposed a great strain on the strength of the Russian people and swallowed up many victims. Disturbances have broken out in the country itself. The evil-minded leaders of the revolutionary movement make insolent attacks on the Holy Orthodox Church and the lawfully established pillars of the Russian State. His Majesty appeals "to right-minded people of all classes to join him in single-minded co-operation in the task of overcoming the stubborn foreign foe and of eradicating revolt at home, and in wise efforts to check internal confusion"; and concludes, "May God send down on the clergy holiness, on those in authority justice and truth, on the people peace, on the laws power, and on faith strength to the consolidation of the autocracy and the welfare of our dear subjects." On Friday afternoon the Tsar addressed a rescript to the Minister of the Interior, in which, after thanking the nobility, Zemstvo Assemblies, commercial associations, and peasant communities for their congratulations upon the birth of an heir, he assured them that the wish of his heart is to co-operate with them in the successful execution of the reforms he announced, and to convene, with the help of God, the worthiest men, possessing the confidence of the people and elected by them, to co-operate with the Government.

### Great Battle in Manchuria.

The great battle which has been proceeding for some days in Manchuria is described as being on an even larger scale than the battle of Liau-yang. On both flanks the fighting has been desperate, and the charges of the Japanese most persistent. The losses on both sides are estimated at 100,000, and it is stated that the Russians have had to abandon their heavy guns on the Sha-ho. The Russian right army fought without ceasing for six days, until compelled by an enveloping movement to withdraw. The battle is still raging.

### New High Commissioner of South Africa.

Following the official intimation of the resignation of Lord Milner, it was announced last week that the office of High Commissioner in South Africa and Administrator of the Transvaal and Orange River Colonies has been conferred upon Lord Selborne. The new High Commissioner, who was born in 1859, is the son of the famous Lord Chancellor Selborne. He entered the House of Commons as Lord Wolmer in 1885. In 1895 he entered the Salisbury Ministry as Under-Secretary of State for the Colonies, serving for some time under Mr. Chamberlain. In 1900 he was promoted to the office of First Lord of the Admiralty. Lord Selborne married in 1883 Lady Beatrix Maud Cecil, daughter of the late Lord Salisbury.

### Changes in the Cabinet.

In consequence of Lord Selborne's appointment as High Commissioner of South Africa, the vacant post of First Lord of the Admiralty has been conferred upon Earl Cawdor. The new First Lord was born in 1847, and sat in the House of Commons as Viscount Emlyn from 1874 to 1885. In 1898 he succeeded his father in the title and family honours. For the last ten years Earl Cawdor has been chairman of the Great Western Railway which, during that period, has shown itself to be among the most enterprising of our railway systems. His great business powers and administrative capacities are believed to admirably qualify him for the office of First Lord of the Admiralty.—On Monday the Prime Minister announced in the House of Commons that Mr. George Wyndham had resigned the post of Irish Chief Secretary. Mr. Wyndham, who is 42 years of age, served from 1887 to 1892 as Private Secretary to Mr. Balfour. From 1898 to 1900 he was Under Secretary for War. In 1902 he became Irish Chief Secretary, from which he now retires in consequence of difficulties arising out of his administration with the Ulster members.

### Three New Bishops.

It was officially signified on Tuesday that the King has approved the appointment of the Bishop of Rochester (Dr. E. S. Talbot) to be Bishop of the new See of Southwark; the Bishop of Adelaide (Dr. J. R. Harmer) to be Bishop of Rochester; and the the Rev. Dr. E. S. Gibson, vicar of Leeds, to be Bishop of Gloucester. The Bishop of Southwark before his appointment to Rochester was vicar of Leeds. Dr. Talbot came to London 10 years ago next October, and his decision to serve as head of the new diocese, rather than as Bishop of the ancient See, was anticipated. The new Bishop of Rochester was born in 1857, and is the son of a Gloucestershire clergyman. He had a distinguished career at Cambridge, and served under Bishop Lightfoot of Durham. In 1890 he was elected to a Fellowship at Corpus Christi College, Cambridge, and in 1895 he went out to Adelaide as second Bishop of the New South Wales diocese. The new Bishop of Gloucester was born in 1848 at Fawley, Hampshire, where his father was rector. He was in turn Chaplain, Vice-Principal, and Principal of Wells Theological College, and in 1895 he succeeded the new Bishop of Southwark as Vicar of Leeds.

### Advances in Wireless Telegraphy.

On Friday, last week, Mr. Marconi, who is to be married this month, lectured to a crowded audience on "Recent Advances in Wireless Telegraphy." He said that recent improvements included means to prevent interference and secure independence of communication, to increase the distance over which communication was possible, and to obtain greater efficiency and accuracy. Amongst a large section of the public at the present time there was a misconception of the term "intercepted," in speaking of messages which were "tapped" by another wire. It was quite true that messages were tapped, or overheard, at stations for which they were not intended, but that did not prevent them from being received distinctly at their proper destinations. Mr. Marconi gave details showing the extent to which his system is used, and expressed his confidence that wireless telegraphy would be able to provide communication between England and her Colonies, and England and America, more economically than the submarine cable. It was gratifying to him that it had already done something towards increasing the comfort and decreasing the perils of those that go down to the sea in ships.

### A Book and its Story.

SIR FREDERICK TREVES' TOUR ROUND THE WORLD.*

There is not one dull page in Sir F. Treves' "account of a commonplace tour round the world," which is the modest sub-title chosen to support the more ambiguous one of "The Other Side of the Lantern. To explain this choice of title the book is prefaced by the following picturesque paragraph:—

"A paper lantern, round and red, hangs under a cloud of cherry blossom in a Japanese village. There is a very familiar flower symbol painted upon one side of it. Some children have crossed the green to see what is on the other side of the lantern. A like curiosity has led to the writing of this trivial book." Trivial in no sense is the record of places, and people, and things met with in India, Burmah, China, Japan, and America, for it is made by one who, while keenly observant, has ever a kindly sympathy for whatever arrests his attention and it is this that makes the book so readable, for the ground gone over is in no sense new, and yet there is a freshness about the impressions that makes them an ever-varying delight to peruse. First impressions of India are given as follows:—

"Possibly the first impression of India, which succeeds the realisation of the strangeness of all things, is an impression of *teeming* life—of the unwonted number of living beings, human and animal, who crowd the land. . . . They crowd everywhere over the length and breadth of the peninsula, for they number two hundred and ninety millions." This multitude of men, women, and children exist in a condition described as one a little below the most meagre comfort and a little above the nearest reach of starvation. "In the towns they hustle one another as they trample along in the dust so that each narrow street is full to its walls. The driver of a donkey has to yell himself hoarse to make way for his beast, and the bullock reaches the lane's end by ploughing his shoulders through the crowd as through a field of maize. From the tower of any walled city a many-coloured stream can be seen moving out of each gate across the plain. The streams are made up of brown-faced men, tramping to be lost on the horizon, tramping to be lost in the town. From sunrise to sundown the muffled sound of their steps never ceases, and at night there is no dark alley without the sleeping figure of the homeless man." Animals appear to mix indefinitely in an Indian crowd and to be as much a part of it as human beings. They wander in and out of the traffic as if they were alone in the desert. To realise the scene, the Bond Street of Delhi is transferred to the Bond Street of London that the reader may imagine; "Camels striding down the road with supercilious disregard of the police, a train of donkeys winding among the hansoms, and goats picking their way between the carriages. A fat grey bull would be dozing on the step of a hatter's shop, a couple of sheep would be nosing among the trifles of a milliner's counter; while a buffalo, laden with a pile of straw, would hustle the frock-coated lounger from the pavement."

Next to the impression of teeming human and animal life comes the realisation that India is a country of intense colour. "Brilliant, crude, and lavish" are the contrasts of light and shade, under the bright blaze of an Indian sun. In recalling this picture of India, Sir Frederick writes as an artist: "A hard, azure sky against which stands out, keenly out, some cocoa-nut palms, and a slate-coloured dome. The dome rises above a white wall, which ends below in a dusty road. It is ever in India the white wall with the sun on it. . . . Crows like beetles look down from the wall. A purple hood for the head, and a scarlet gown, a bright green turban with an amber cloak, an orange-tinted tunic and a yellow scarf, a naked brown boy, and a man clothed all in white, make up the ever-changing eddies of colour in the street. The light that beats upon all is blinding, while the shadows by the walls are lit up with gleams of brass vessels and the silver bangles on the women's feet."

Although the English in India form an exceedingly small company England is the only Western Power that has maintained a unique influence over the dealing of its ninety millions of people. The Portuguese were the first to set foot in the country, so long ago as 1498, with the arrival of Vasco da Gama; their possessions are now represented only by three small settlements. The Dutch, in spite of valiant endeavours to absorb the mighty peninsula, have now no soil there. The Danes, Swedes, and Prussians established trading companies but the memory of them alone remains. The French have played a determined part in an attempt at colonisation but the French flag to-day flies only over five towns. On British rule in India Sir Frederick writes:

"The governing of India is a wondrous thing to contemplate; wonderful to reckon by how few it is done, with what apparent ease and small parade of power; wonderful to see how difficulties have been moulded into gains, how prejudices have been turned to good account, and how strong bricks have been made from uninviting straw. Above all are to be admired those broad principles of justice, honesty and kindness which are at the foundation of British rule."

The beautiful photographs taken by the author of various places of interest, and exquisitely produced, add to the attraction of the book. In passing we must notice one which faces the title-page, bearing the touching inscription of "The Garden of the Unforgotten." It shows the entrance gates to the shrine of the greatest of the Chisti Saints, to memorials to past rulers, and other people of native distinction.

Among so much that is interesting it is difficult to choose passages in illustration of Sir Frederick's powers of acute observation in the limited space at disposal. But the following extracts should be of interest to readers of The Hospital, in reference to the Japanese and their talent as surgeons.

"To the stranger from the Occident the Japanese are a somewhat inscrutable folk, bristling with the unexpected, full of surprises and apparent contradictions. They appear to be easy and unbusiness-like. . . . and yet these people are conducting a war as no war has ever been carried on before. . . . The Japanese are acutely imitative, but to this must be added that they have improved whatever they have copied. So far as there is any character in modern surgery, that of Japan is German. It is, however, being improved upon in countless details. The Japanese surgeon is no longer a servile imitator. He is introducing into his methods the results of his own ingenuity. Many features which in Europe are of the latest suggestion have already been anticipated in Japan. There is every probability that the Japanese school of surgery will become a great school, for the native of Japan has qualities which are excellent in the making of a surgeon; he is not troubled by 'nerves,' he is infinitely patient, fastidiously clean, as well as most neat and dexterous with his hands. Moreover, he has a love of ritual as well as of precision in ritual, and in the prosecution of antiseptic surgery this counts for much." We hope that the great demand for "The Other Side of the Lantern" will lead to the issue of a cheaper edition, and so make it accessible to the general public.

* "The Other Side of the Lantern." By Sir Frederick Treves. (Cassell and Company. 12s. net)

### Notes and Queries.

**REGULATIONS.**

**The Editor is always willing to answer in this column, without any fee, all reasonable questions, as soon as possible.**

**But the following rules must be carefully observed :—**

**1. Every communication must be accompanied by the name and address of the writer.**

**2. The question must always bear upon nursing, directly or indirectly.**

**If an answer is required by letter a fee of half-a-crown must be enclosed with the note containing the inquiry.**

*Fund for Sick or Aged Nurses.*

(170) I should be glad to know whether there is such a thing as a fund for sick or aged nurses.—*Nurse M.*

The Royal National Pension Fund for Nurses is the best provident fund. Write to the Secretary, 28 Finsbury Pavement, E.C.

*District Nurse.*

(171) I have been a district nurse for 18 months, and had three years' training in Guy's Hospital. I have the L.O.S. certificate, and should like to be an inspector of midwives and health visitor. Do you think I should stand any chance? Also will you give me the address of the Sanitary Institute, and is their examination very difficult?—*B. B.*

Your qualifications should give you a good chance of becoming an inspector of midwives, though in some places they are appointing medical women to these posts. All particulars of the Sanitary Institute, with fees, lectures, etc., can be obtained from the Secretary, Sanitary Institute, Margaret Street, Westminster, S.W.

*Address.*

(172) Will you kindly tell me the address of the Central Midwives Board, as I wish to register?—*F. A. C.*

6 Suffolk Street, Pall Mall, S.W.

*Cap.*

(173) Will you kindly tell me if the Army nursing cap may be worn in private nursing. I do not belong to the Army nurses?—*Anxious.*

In private nursing you can, of course, wear any cap you like, provided you do not belong to an institution and have to wear a special uniform. But it would be very bad form indeed for you to wear an Army nursing cap.

*Insurance for Nurses.*

(174) Will you kindly give me the address and name of the insurance and sick fund for nurses under Royal patronage?—*Pension.*

The Royal National Pension Fund for Nurses, 28 Finsbury Pavement, E.C. Write to the secretary.

*Canada.*

(175) A fully-qualified nurse is going to Canada next month. Will you kindly tell her the best way of procuring cases on arrival. Can she enter a private home? Is there a good opening in Gault, Ontario, or would New York be better?—*L. W.*

Write to the Secretary, Colonial Nursing Association, Imperial Institute, S.W.

*Epileptic Institutions.*

(176) 1. Is there any institution which would receive a girl of 18, epileptic, and a little backward in most things? Payment could be made of 10s. weekly; home preferred. 2. What steps must I take, and how, to get a boy taught to learn the business of a chemist, and what is the average wage of a qualified assistant to a chemist?—*H. M. W.*

1. Write to the Secretary of the National Society for Employment of Epileptics, 12 Buckingham Street, Strand, W.C.; also to the Secretary of the Meath Home of Comfort for Female Epileptics, Westbrook, Godalming. 2. Your second query is outside this column as it does not bear, either directly or indirectly, upon nursing. See rules above.

*Midwifery.*

(177) Will you kindly inform me if it is possible to get free training for midwifery in London at some hospital or school where they have the L.O.S. examination?—*A. B.*

Write to the Secretary, Rural Midwives Association, 47 Victoria Street, S.W.; also to the Midwives Institute, 12 Buckingham Street, Strand, W.C.

**Handbooks for Nurses.**

| | Post Free. |
|---|---|
| "A Handbook for Nurses." (Dr. J. K. Watson.) ... | 5s. 4d. |
| "The Nurses' Dictionary of Medical Terms." ... ... | 2s. 0d. |
| "Massage" (Swedish.) ... ... ... ... ... | 2s. 6d. |
| "Surgical Ward Work and Nursing" ... ... ... | 3s. 10d. |
| "A Complete Handbook of Midwifery." (Watson.) ... | 6s. 4d. |

Of all booksellers or of The Scientific Press, Limited, 28 & 29 Southampton Street, Strand, London, W.C.

### For Reading to the Sick.

PRAISE IN PAIN.

I sought to praise Thee, but my heart
Went heavily along;
It seemed too weak with sorrow's smart,
To lift itself in song.

I sought to count Thy mercies o'er,
To view them one by one,
But sighed o'er what may be no more,
Chief blessings that are gone.

Till I am brought to worship now,
E'en for this very grief;
To praise the mercy with which Thou
Hast kept back all relief.

*C. M. Noel.*

We can only say with ourselves, "If Thou, Lord, wilt be extreme to mark what is done amiss: O Lord, who may abide it?" Conscious to ourselves of God's manifold gentleness, we must needs be gentle too.

"His way"! Sanctifying work equally requires a gentle hand. We need to be gentle even with ourselves. "The wind bloweth where it listeth"; let us think of the manifold ways in which grace comes to us. There are many forms of life and many forms of excellence in the world. Solomon's temple is reared by a variety of work and different kinds of workmen. Some have to quarry out the stone in the remote mountain side; some have to shape it and carve it for its place, far away from the excitement of finished work; some have to hew timber in Lebanon; some to dig foundations at Jerusalem; some to build, and some to finish.

Spiritually, gentleness is the abiding sense of the honour and dignity of our Heavenly origin. And so this fruit unfolds itself, it follows hard upon long-suffering; being a sort of constant realisation of our Father, coupled with a feeling of reverence, tenderness, self-restraint, and respect, which comes from habitually living in the presence of God. "Such as are gentle, them shall He learn His way." "Thy gentleness hath made me great."

"Thy gentleness hath made me great." *Gentleness*, has made its way to the crown; and he who has proved his gentle birth as a son of God, here in the rough campaign of life, by his reverence and quietness, will hereafter be received into his birthright. As it is written, "Blessed are the peace makers; for they shall be called the children of God."—*Canon Newbolt.*

Resolved that I my part fulfil
In what I know to be Thy will;
Resigned, in that I will rejoice
Which is in Thy dear choice;
If Thou wilt only in Thy love
Prepare me for Thy house above.
Resolved in Thee to do my best,
Resigned to leave the rest
With Thee, and so contented be,
With what Thou thinkest best for me.

*Anon.*

## Figures and Tables

**Figure f1:**
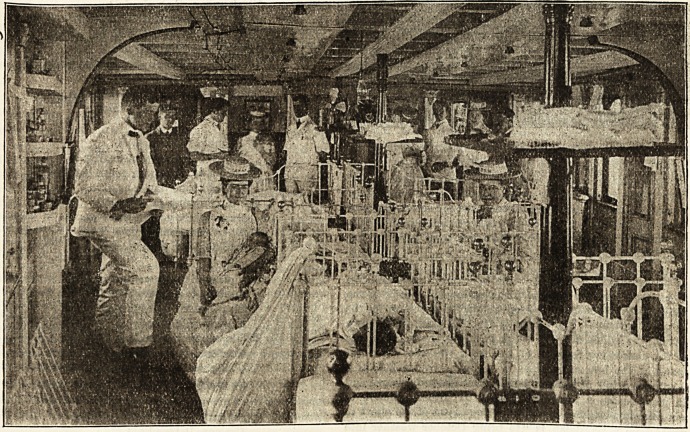


**Figure f2:**